# Musicians, the music industry, and suicide: epidemiology, risk factors, and suggested prevention approaches

**DOI:** 10.3389/fpubh.2025.1507772

**Published:** 2025-03-07

**Authors:** George Musgrave, Dorian A. Lamis

**Affiliations:** ^1^Institute for Creative and Cultural Entrepreneurship, Goldsmiths University of London, London, United Kingdom; ^2^School of Medicine, Emory University, Atlanta, GA, United States

**Keywords:** musicians, occupational health, music industry, suicide, risk assessment, prevention

## Abstract

Evidence suggests that popular musicians are an at-risk occupational group for suicide, with the deaths of famous musicians in the ‘27 Club’ reinforcing culturally powerful notions of musicianship and early mortality. This cross-disciplinary paper advances our understanding of the factors that may increase the risk for suicide among musicians and offers clinical recommendations around screening and prevention. First, we synthesise extant literature on suicide risk among musicians from around the world, including emerging evidence from Korea, and evaluate some of the methodological challenges presented in the analysis of suicide data on musicians. Second, given the lack of musician-specific forms of suicide prevention intervention, we draw on the Zero Suicide Framework and apply this schematic to musicians and the wider music industries, analysing the latest evidence on suicide screening, assessment, and prevention to develop best practices in this at-risk population. In doing so, we offer a comprehensive and clinically relevant overview of this most tragic of cultural affinities to improve strategies to prevent this devastating and all too frequent feature of musical life.

## 1 Introduction

Better understanding the mental health and wellbeing of musicians who pursue a career within popular music,^1^ as well as the development of effective mental health interventions for these musicians, has become a subject of increasing scholarly interest among researchers studying a range of popular music genres and informed by a variety of interdisciplinary perspectives (1–6). Among the general public, perhaps the most well-known manifestation of the relational dynamic between creative workers and poor mental health concerns tragic early death, in particular the highly publicised suicides of famous musicians. From *Nirvana’*s Kurt Cobain, *Linkin Park’s* Chester Bennington, *Joy Division’s* Ian Curtis, and country music singer Mindy McCready, to Keith Flint of *The Prodigy*, Electronic Dance Music (EDM) DJ Avicii, and K Pop stars Goo Hara, Sulli and Moonbin, these tragedies serve to culturally reinforce this most devastating of apparent affinities. Of particular infamy, and afforded an almost ‘mythical’ status (7), are a group of musicians all of whom died at the age of twenty-seven by suicide and other forms of violent death known as the ‘27 Club’ (8). Although empirical evidence does not highlight a spike in death at the age of twenty-seven for musicians (9), this ‘club’ has done much to reinforce culturally powerful notions vis-à-vis musicianship (and creativity more broadly) and early mortality, and problematic conceptualisations of artists as being inherently ‘tortured’ (10).

Moving beyond this unhelpful and potentially harmful romanticisation of suicide, this paper seeks to engage with the latest objective epidemiological evidence on suicide among musicians. For example, routine mortality data for England from 2011 to 2015 shows that the occupational groups with the highest risk of suicide were: construction workers; building finishing tradespeople; agricultural workers; musicians, actors and entertainers; and carers. Those working as musicians, actors and entertainers were the highest risk group within the occupational group defined as culture, media and sport occupations, in which overall the suicide rate for males was 20% higher than the male average and for females was 69% higher (11).^2^ Likewise, data from the United States (12, 13) – where the total age-adjusted suicide rate in 2022 was 14.2 deaths per 100,000 standard population (188) - shows that the occupational group categorised as ‘Arts, Design, Entertainment, Sports and Media’ (of which musicians of all genres form part) had the highest female suicide rate of all occupational groups in 2012, 2015, and 2021, and among males, Sussell et al. (13) found that musicians, singers, and related workers (138.7 per 100,000) had the third highest suicide rate among major occupation groups only behind logging workers (161.1 per 100,000) and agricultural and food scientists (173.1 per 100,000). Statistics such as these necessitate the kind of detailed attention on the relationship between musicians and suicide we offer in this paper.

Informed by a desire both to understand suicide among popular musicians – that is, those working in non-classical genres such as pop, rock, dance, rap/hip hop, EDM, etc. - and improve clinical recommendations around screening and prevention, this cross-disciplinary position piece based on a review of the literature brings together interdisciplinary fields of scholarly inquiry to address two questions. First, based on the evidence and theory, what are the factors likely to increase the risk of suicide in musicians? Secondly, what does current evidence from the study of suicide screening, assessment, and prevention (encompassing referral for care, treatment and follow-up) teach us concerning the application of best practices for musicians, and what are the challenges for those who work with and care for these musicians in their personal and professional networks? In answering this second question, we employ the Zero Suicide Framework and, informed by one author’s clinical experience, suggest how it might be adapted for musicians and those working in the wider music industries. We hope that addressing these questions will both advance our understanding of the cultural, sociodemographic and clinical factors that may increase the risk for suicide among musicians, and inform the work of suicidologists eager to better develop more effective methods of suicide intervention as has been observed in a range of other industries and occupations which have also been found to be high-risk [e.g., construction workers (14, 15)]. In bringing two authors of different disciplinary expertise together – one specialising in the music industries and musicians’ health, and the other specialising in suicide prevention specifically – we offer the first paper of its kind to both synthesise the existing evidence on musicians and suicide *and* make clinical recommendations for strategies to prevent this most tragic feature of musical life.

## 2 Musicians and suicide risk

### 2.1 Prevalence and risk factors

Extant research suggests that broadly-defined ‘artists’ (an occupational category which includes musicians working in both classical and popular traditions, alongside other creative workers) are an at-risk group for suicide. Occupational data over the past eighty years predominating from the Global North including Germany (16), the United States (13, 17–19), England (20), England and Wales (21), Britain (22), Australia (23), and across various other countries (24) have all shown musicians performing in various genres (particularly younger musicians) to have an elevated risk of suicide compared to other occupations. While studies such as these on ‘artists’ have often shown the sub-category of musicians to be less likely to die by suicide than other creative occupations – such as poets, or visual artists - occupational research more broadly demonstrates that musicians have an elevated suicide risk, alongside other creative workers as well as other forms of employment, notably construction workers.

Elevated incidence of suicide among musicians have been conceptualised as resulting from high levels both of depression (25–28) and substance use/abuse within this group (29–31). With reference to these two traits, scholars have attempted to ascertain whether or not being a career musician might attract those with specific orientations or temperaments - with debate among those who suggest musicians are a unique group vis-à-vis temperament and the prevalence of mood disorders and subsequent potential links to suicide (32–34) - and those who reject this hypothesis (35). Others have highlighted psychosocial music industry working characteristics which might encourage or facilitate risk-taking behaviours, and thus may heighten the exposure to suicide risk (19, 36). These two hypotheses concerning the predispositional and the psychosocial are likely connected. However, the latter suggestion that working in the popular music industries presents *particular* risk factors for suicide is supported by evidence showing high levels of suicidality not only in musicians but also among the wider music industry profession. Work on entertainment professionals in Australia (including the music industry) found suicidal ideation over a lifetime to be more than six times higher than the general Australian population (37). Moreover, recent research on music industry touring professionals and artists found that, together, their prevalence of suicidality - measured using The Suicidal Behaviours Questionnaire-Revised (38) - was estimated to be more than five times higher than the general population in the United States (39, 40). These findings point to, perhaps, something distinct about the working conditions of the popular music industry as potentially being impactful.

What, then, are some of the suicide risk factors for musicians? In the first instance, gender is key in the empirical data. Although males have been found to be at greater risk of suicide than females in data from around the world (41) – albeit with females attempting suicide more frequently [known as the gender paradox in suicide (42)] - work on musicians shows a more nuanced picture. As aforementioned, epidemiological data from the United Kingdom (11) and the United States (13) reveals female musicians die by suicide at elevated levels compared to females in the general population. In a study specifically on popular musicians’ deaths (*N* = 13,195), Kenny and Asher (43) note: “unlike females in the general population, female gender did not bestow any protection against early death or manner of death by suicide, homicide or accident compared to male popular musicians.”

In a comprehensive meta-analysis of risk factors for suicidal thoughts, behaviours and deaths, Franklin et al. (44) identified sixteen broad groups of risk factors, many of which appear to be prevalent among musicians based on the limited and emerging evidence in this field. The top risk factors that were found to be the strongest predictors across the suicide-related outcomes were:

- *Prior suicidal ideation and attempts*: A survey of musicians by Berg et al. (45) found 17.4% of respondents had a history of at least one suicide attempt, although larger and more representative datasets are limited.
- *Abuse history* (of any kind; sexual, emotional, physical): There is some evidence to suggest that abusive practices – notably bullying, harassment and sexual abuse - are prevalent in the popular music industry (46). Work by Jones and Manoussaki (47) in the United Kingdom, for example, suggested sexual abuse and misogyny to be rife in the music industry, and, as per the title of their report, ‘completely entangled in its fabric’. This has been further emphasised by the recent UK House of Commons Women and Equalities Committee (48). Musicians have been documented as suffering from racist abuse too (49). Finally, it has been suggested that many musicians have suffered from various forms of trauma or adversity which has inspired their music-making (50), although it is hard to be precise about the numbers impacted in this occupational group.
- *Depression/anxiety diagnoses*: Work by Gross and Musgrave (51, 52), Vaag et al. (5), Loveday et al. (53), and Musgrave et al. (54) has shown these conditions to be high among musicians relative to the general population, albeit these studies rely on self-selecting samples with only work by Vaag explicitly making comparisons between musicians and the general public in their study in Norway.
- *Previous psychiatric hospitalisation*: While not evidenced directly, data from Norway has suggested musicians use psychotherapy and psychotropic medication more than the general population (5) and the nature of musicians seeking psychiatric help has been considered in the work of van Fenema et al. (184).
- *Low socioeconomic status*: Financial precarity and low earnings are a well-understood and endemic feature of musical work for popular musicians (45, 185, 186).
- *Stressful life events*. Work on musicians has suggested psychosocial features of their working lives to engender significant stress, and that features of musical career development might be health-threatening in multiple ways alongside, and in addition to, the previous risk factors mentioned above [see systematic review by Willis et al. (55)]. These include: irregular schedules impacting sleep and circadian rhythms (2), financial instability (56), isolation and loneliness (183), relationship strain (57), physical health problems (e.g., musculoskeletal) (58), vulnerability derived from sharing close personal experiences on social media (59), stresses associated with touring (60), and the prevalence of eating disorders (61) and performance anxiety (62), both of which have been suggested might be linked to high levels of perfectionism among this population (63).

There are other important sources of stress in musicians’ lives too revealed through the tragic cases of musicians who have died young. For example, Jimi Hendrix, who died at the age of twenty-seven, told a reporter a few weeks before his death: “The moment I feel that I do not have anything more to give musically, that’s when I will not be found on this planet, unless I have a wife and children, because if I do not have anything to communicate through my music, then there is nothing for me to live for. I’m not sure I will live to be twenty-eight years old” (64), demonstrating how deeply intertwined musicianship becomes with a musician’s identity (65). Likewise, EDM DJ Avicii, who died by suicide at the age of twenty-eight, is seen in the documentary *Avicii - True Stories* (66) expressing upset over his exhausting touring schedule, and can be heard prophetically saying: “I have told them this: I will not be able to play anymore. I have said, like, I’m going to die. I have said it so many times, and so I do not want to hear that I should entertain the thought of doing another gig.”

A factor analysis of the Musician Occupational Stress Scale (MOSS) reveals eleven key factors of stress including performance (and performance-related) anxiety, work over/underload related to travelling, career development, poor physical work conditions, effects on social and family life, conflicts within a band, and working relationships (190). For Franklin et al. (44) intensive occupational stressors (such as these faced by musicians) may have the capacity to engender parasuicidal behaviour and even suicide itself, and the relationship between lifestyle behaviour, psychological symptoms, and suicide risk is well documented (67).

Finally, we turn to genre. Suicide has been found to be lowest in gospel musicians (68), potentially being mediated by religiosity. Jazz musicians have been afforded particular attention (69), and suicide has been shown to be most prevalent in the genres of punk (68), country, rock, and particularly (heavy) metal (36). The genre of metal has been singled out in particular in the literature as presenting specific risk factors for suicide, although much of this work has focused on *listeners* of metal music and other subgenres (e.g., ‘emo’), particularly among adolescents and teenagers, as opposed to *musicians* themselves (70, 71, 191). However, this relationship is complex; Baker and Brown (72) and Howe et al. (73) recognise the subcultural community offered by the genres in question as serving as a protective factor against suicide. More recently, a genre referred to as either ‘emo [emotional] rap’ or ‘Soundcloud rap’ has attracted controversy for lyrics which often heavily-reference suicide or suicidal ideation (74, 75), with one prominent New Orleans-based group even named Suicideboys. However, despite evidence that what might be considered ‘problem music’ (such as this given lyrical themes) might lead to self-injurious thoughts and behaviours (76), there is no scholarly evidence on increased suicide risk for either performers or listeners of this genre (or group of genres) as this has not yet been investigated.

Within genre, we would also highlight substance use as a risk factor. Musicians as an occupational group have been seen to use illicit substances for various social, cultural and artistic/creative reasons (77, 78), as well as exhibit higher levels of problematic drinking compared to non-musicians (79). Several studies have suggested genre to be a moderating factor e.g., Howard Becker’s (187) classic sociological work on marijuana use among jazz musicians, Singer and Mirhej’s (80) ethnography of marijuana and heroin use in jazz too, to more recent empirical work suggesting substance use among 226 New York-based musicians was associated more with certain genres (e.g., rock, rap/hip hop) than others (e.g., pop and jazz) (81). Both alcohol and drug use disorders have been shown to be associated with suicide in empirical studies (82), and more work on this potential association among musicians is needed.

One genre worthy of investigation concerns ‘K Pop’ – a style of popular music emanating from South Korea (henceforth, Korea) with a specific aesthetic orientation and emphasis on performance (83). Contemporary media reports have done much to draw the wider public’s attention to a range of high-profile suicides among young ‘idols’ (as they are known) within this particular scene, such as Moonbin, Jonghyun, Sulli and Goo Hara (84–86). K Pop warrants attention in particular given that the vast majority of research into musicians and suicide, and indeed into musicians and mental health more generally, is overwhelmingly based on music careers from the United States of America and/or Europe. Thus, evidence from Korea offers a new cross-cultural music industry context in which to explore this phenomenon.

Scholarly evidence is relatively nascent in this population and at present there is no available data (that we are aware of) on whether the prevalence of suicide is higher among Korean musicians than the general public in Korea, which has one of the highest suicide rates in the world (87). However, research does highlight a number of culturally specific risk factors among this population of musicians. For example, K Pop fandom has been characterised as obsessive (88) with one of the few scholarly works focusing on K Pop and suicide employing a Durkheimian lens to foreground the impact of cyberbullying by fans and the marginalisation this can create among K Pop ‘idols’ as potentially leading to suicide (89). While online parasocial relationships between fans and K Pop musicians have been shown to improve wellbeing among fans themselves (90) they have been conceptualised as placing undue strain on musicians, with Mercier (91) highlighting ‘toxic practices in K-Pop fandom’. Additionally, K Pop ‘idols’ often undergo intense regimes of early childhood training in practices such as singing and dancing (92), and later have to endure gruelling schedules vis-à-vis touring and media appearances (93). However, some researchers have highlighted that these challenges might not be unique to these musicians per se but are instead amplified reflections of trends for high-pressured, socially-prescribed perfectionism in spheres of work and education in Korea more generally (93, 94). These findings underscore the need to understand the relationship between musicians and suicide as part of the mutual interaction between music industry working practices and the societies in which these practices manifest [see (95)].

### 2.2 Methodological considerations

Although the risk of suicide appears to be elevated among musicians, some notes of methodological caution are warranted. First, while the focus in this paper concerns musicians working in the popular music industries specifically, many studies of musicians and suicide (particularly historical studies) have drawn upon famous examples from classical music too, such as Otto Mahler. Though there are overlaps between these two fields (e.g., financial precarity), there are salient differences too, and thus discerning the impact of the contemporary popular music industry is challenging. Secondly, terminological and methodological debates regarding how to identify and appropriately categorise a death as suicide in musicians are complicated. For example, work on morbidity and mortality among popular musicians by Kenny (68) notes that quantitative studies which seek to understand patterns and prevalence of suicide in musicians can be constrained by how deaths are classified by coroners, impacting scholarly practices of coding. Citing the contrasting examples of INXS’s front-man Michael Hutchence and the singer Amy Winehouse, Kenny notes that despite high risk, and even parasuicidal behaviours by both musicians, their deaths were recorded differently. Hutchence’s death was recorded as suicide by asphyxiation despite his wife’s insistence that this was likely accidental auto-erotic strangulation, whereas Winehouse’s death was recorded as alcohol poisoning despite her hazardous drinking behaviour and high risk of death being communicated by her physician. Likewise, the English singer-songwriter Nick Drake’s antidepressant overdose was categorised as suicide, as was the American punk rock singer Darby Crash’s heroin overdose, whereas the heroin overdose of Howie Epstein (the bass player with Tom Petty and the Heartbreakers) was not. Kenny suggests that examples such as these have resulted in many suicides being mis-classified as accidental overdoses.

Another methodological consideration concerns the preponderance of biographical studies of suicide among musicians (i.e., studies which focus on one specific musician’s suicide, or on suicide data relating only to particularly famous musicians). Partridge (96) refers to this as ‘artist-centred approaches’ to the study of suicide. The suicides of those in the ‘27 Club’ form the core of this literature, with particular focus afforded to the suicide of Kurt Cobain in 1994. Notable in the literature is the extent to which the ‘Werther effect’ (i.e., copycat suicides)^3^, might have followed Cobain’s death given the media coverage which surrounded it (97) – with scholars suggesting in empirical work that this was not the case (98, 99) – and linguists seeking to derive insights from Cobain’s apparent suicide note (100, 101).

Similarly, many empirical studies exploring suicide and other forms of mortality among this population also draw on biographical data of well-known musicians [e.g., (24, 29, 33, 102–104)]. However, given this, it can be difficult to account for fame – or ‘eminence’ – as a core variable given that these studies tend to focus only on famous musicians as opposed to those who are unknown. The problems inherent in the biographical approach are noted by Pavitra et al. (35) who remark: “Despite the robustness of the statistics, they must be considered cautiously. Diagnoses have often been made posthumously using loose and inconsistent diagnostic criteria. In many cases the validity of a diagnosis of mental illness, alcoholism or suicide cannot be verified. In addition, solid statistical evidence of the extent of mental illness, alcoholism and suicide in the general population is still far from precise. Finally, it is possible that the creative persons with dramatic lives and early deaths are more likely to become eminent and have biographies written about them.” However, as Kenny (68) notes, given the challenges in identifying popular musicians as an occupational category, and subjectivities regarding the attribution of ‘professional’ status (53), it is hard to get a valid and representative dataset on the number of musicians working in genres of popular music in any one territory from which to sample and conduct quantitative analysis. This perhaps explains the extensive use of biographical material to study this subject.

It is also worth noting that biographical material can often provide rich, textured, qualitative insight which can offer important perspectives alongside other kinds of studies from this field of inquiry. Examples include journal entries (105) or lyric analysis, as in the case of the suicide of the rock and roll singer Chris Cornell (106). Indeed, lyric analysis has emphasised elevated levels of distress connected to deaths by suicide among ‘27 Club’ musicians (107) and rappers (108), findings not however corroborated by Gunn and Lester (109) who found no changes in lyrical content over time, suggesting instead what they refer to as “the uniqueness of the psychological states leading up to suicide.”

Overall, despite important methodological challenges and debates vis-à-vis causality, it seems reasonable to conclude, simply, that popular musicians do appear to be an at-risk occupational group for suicide. The next part of this paper will draw on clinical experience to explore how this risk might be mitigated.

## 3 Suicide prevention among musicians: applying and adapting the Zero Suicide Framework

Having ascertained that popular musicians seem to be an at-risk population, the next logical step is to reflect on what might be done to prevent such tragic loss. In general, there are evidence-based strategies that have been shown to be effective in identifying those most at risk, assessing risk level, and intervening to reduce suicidal ideation and behaviour (110), yet none of these approaches have been developed or evaluated among musicians specifically in peer-reviewed scholarship. Therefore, below, we use a seven-part approach developed by the Zero Suicide Framework (ZSF) (Table 1). This is a systems approach to suicide prevention formulated following recommendations made by the National Alliance for Suicide Prevention (111). Although some have reasonably evaluated the suitability of a ‘zero’ suicide approach given the dangers of blame or cultures of guilt in the use of such terminology [e.g., (112)] the ZSF has been shown to effectively reduce suicide attempts (113, 114). However, it is important to acknowledge that not all suicides are preventable, and indeed some bereaved families struggle with perceived self-blame for not having done enough to prevent a suicide. Key to intervention is to identify risk factors and mitigate them accordingly. To this end, we will delineate each element of the ZSF and reflect on how these elements of suicide care might be adapted to the specific circumstances of musicians. It is also important to note at the outset that the approaches outlined below are not based on empirical evidence specific to musicians or other entertainers, nor have they been evaluated in peer reviewed scholarship among this population. In this respect, this section represents the views of the authors, one of whom is a clinician specialising in suicide prevention, and therefore constitutes (drawing on the language of the National Institute for Health and Care Excellence) ‘expert opinion’ as opposed to Level 1/2/3 evidence.^4^

**Table 1:** The Zero Suicide Framework and goals adapted for musicians [table adapted from Turner et al. (114)].

ZSF element	Goal	Music industry/musician-specific adaptation
Leadership	Create a leadership-driven, safety-oriented culture committed to dramatically reducing suicide among people under care. Include people with lived experience in leadership and planning roles.	Music-industry awareness campaigns for suicide prevention and campaigns drawing on musicians with lived experience; promoting accessible and affordable healthcare for musicians.
Training	Develop a competent, confident and caring workforce.	Suicide gatekeeper training (121); empowering those who work with musicians, e.g., to ask about suicide directly.
Identify	Systematically identify and assess suicide risk among people receiving care.	Applying risk factors from Franklin et al. (44) to musicians; understanding warning signs.
Engage	Ensure every person has a pathway of care that is both timely and adequate to meet their needs. Include collaborative safety planning and restriction of lethal means.	Columbia Suicide Severity Rating Scale Screen Version (143); Safety Planning Intervention (146); focus on substance use as means for musicians in particular, e.g., lethal-means safety counselling (151), Counselling on Access to Lethal Means (154)
Treat	Use effective, evidence-based treatments that directly target suicidality.	General: Dialectical Behaviour Therapy (157); Cognitive Therapy for Suicide Prevention (158); Collaborative Assessment and Management of Suicidality (159). Musician specific-treatments may be derived from those focused on musician-specific risk factors, e.g., depression [Cognitive Behavioural Therapy for Suicide Prevention (161)] and perfectionism (165).
Transition	Provide continuous contact and support, especially after acute care.	Transition musicians through care with warm hand-offs through providers, especially when providers may change while musicians are touring; caring contacts (173); appointment reminders and bridge appointments important if touring.
Improve	Apply a data-driven quality improvement approach to inform system changes that will lead to improved outcomes.	Data must be collected in this high-risk and vulnerable group of individuals to inform the development of interventions addressing the unique needs of musicians; interventions need to be evaluated and refined to ensure the efficacy of the treatment approach.

### 3.1 Element 1: leadership

“Lead system-wide culture change committed to reducing suicides” (115)

It is crucial to reflect on how a ‘healthier’ music industry might be cultivated and nurtured. As suggested in the discussion above, it is difficult to disentangle suicide among musicians from socio-political norms in wider society and cultural working practices in the music industry. In this respect, mental health education for music industry leaders or those in positions of influence, which includes increasing the awareness and understanding of mental health within the music community and an emphasis on the psychosocial challenges of developing a career as a musician, is a critical component of addressing suicide risk. Public awareness campaigns for suicide prevention should emphasise help-seeking, reduce stigma, encourage positive behaviour change, provide information of available resources, and suggest effective treatments and support (116). This can even be done in song form; work by Niederkrontenthaler et al. (117) showed how a track by rapper Logic entitled ‘1-800-273-8255’ (the phone number of Lifeline) led to an increase in calls and a reduction in suicide among the general population (not musicians specifically), highlighting a paradox that while musicians themselves are an at-risk group, *music* can be an effective tool to prevent suicide. Furthermore, several organisations within the popular music industry (e.g., Backline, The SIMS Foundation, Nuçi’s Space, Man Down Programme, and The Christopher Meredith Foundation among others) have launched campaigns to destigmatise mental health, raise awareness, and educate community members of the serious problem of suicide among musicians, showing leadership in this area. Non-music-specific organisations have also partnered with musicians with lived experience of suicide, exemplified in recent work in the United Kingdom by CALM (Campaign Against Living Miserably) partnering with singer-songwriter Nadine Shah who bravely publicly discussed her suicide attempt (118). These are important steps forward towards raising awareness and reducing stigma.

More broadly, an obvious strategy for preventing suicide is ensuring that all individuals working in the music industry have access to affordable and accessible mental healthcare services, and lobbying for this should be a key area of focus for influential music industry stakeholders and leaders. Ensuring that leaders (e.g., major record label executives, senior leaders in major live music organisations) cultivate partnerships between music organisations and mental health professionals can ensure that musicians have the support they need, including counselling, therapy, and pharmacological services. Engaging musicians and other professionals in the industry along with their families in open conversations about mental health and suicide prevention should reduce stigma and help them overcome barriers to accessing affordable and high-quality treatment. Given that popular musicians often do not have a consistent income (and in countries with private healthcare provision such as the United States may lack access to quality health insurance), it is important that music industry leaders work closely with musicians to connect those working in the music industry with access to mental health professionals who have experience treating this population, who are familiar with their concerns, and who can do so affordably (1, 37). In addition, fostering a sense of community among musicians through supportive online and in-person networks may provide a safe space for sharing experiences, seeking advice, and offering encouragement. Previous studies [e.g., (119, 120)] have demonstrated that peer support groups improve mental health outcomes and quality of life, particularly among individuals who belong to the same social group and have similar interests, such as musicians, and thus the launch of peer support groups for musicians represents a promising development. For those in more acute need, the launch of music-industry specific telephone helplines (e.g., by Music Minds Matter, Support Act, Music Support and others) likewise are a vital part of the intervention landscape with respect to suicide [see (182)].

### 3.2 Element 2: training

“Train a competent, confident, and caring workforce” (115)

Gatekeeper training for music industry professionals is an important step. This is one example of a program that not only raises awareness but educates people on how to recognise a person in crisis, identify behavioural warning signs of suicide, and refer the suicidal individual to appropriate services (121). It has been shown to be effective in enhancing the skills, attitudes, and knowledge of gatekeepers who are defined as people who have primary contact with those at risk for suicide (122). Thus, providing gatekeeper training to everyone working in the recorded music industry and more broadly defined music industries working in a range of roles where they might work alongside musicians (from managers to those working at record labels, booking agents and beyond) to encourage critical professional reflection on where music industry practices might engender risk factors for some musicians should be considered as part of a multifaceted strategy. Situational-related measures such as gatekeeper training, combined with wider public awareness of the challenges faced by musicians in their work, are all, we would suggest, key.

Those who develop careers as musicians are embedded in broad, complex and entangled relational networks of those who support their careers both emotionally and professionally (e.g., family members, teachers, mentors, musical collaborators). For those at certain career stages this network might additionally include a manager, a live booking agent, an A&R (Artist and Repertoire) at a record label or publisher, a tour manager, a PR (Public Relations) agent, and beyond (123, 124). In this context, therefore, improving the understanding of suicide in musicians’ support networks is also likely to be crucial in suicide risk prevention. For example, adding to the large body of research consistently demonstrating that social support is a protective factor for suicide across populations (125), in a study among workers in the entertainment industry results indicated that social support served as a buffer against suicidal behaviour in individuals who are experiencing poor mental health (126). Therefore, empowering those who work alongside musicians to recognise suicide warning signs (see below on ‘Identification’ for more) might be instructive.

Finally, where those around a musician are concerned about suicide risk, evidence suggests that it is critical to directly ask about current suicidal thoughts and previous suicide attempts, and therefore forms of training and support which can aid these conversations is important. Although a reasonable concern, numerous studies [e.g., (127–129)] have demonstrated that a direct suicide inquiry does not have an iatrogenic effect, such as leading to an increase in suicidal ideation, and indeed may even reduce rather than increase suicidal ideation (130). However, it is crucial to acknowledge that mental health and suicide are topics many working around musicians are aware of but feel ill-equipped to handle or ask directly about in this manner. Early mortality is often understood as a serious matter which the managers of musicians, for example, have been shown to care deeply about (131), but that it is perhaps best left to experts such as clinicians. Indeed, on the one hand, one might argue that it is not reasonable or fair to ask everyone who works closely with musicians of this kind to become mental health professionals, and the question of who has a legal or moral ‘duty of care’ for musicians is a vexed issue [see (52)]. On the other hand, some have suggested record labels – following the death of Amy Winehouse (132) – or management companies – following the suicide of Avicii (133) – might have an obligation to do more in this respect. Relatedly, so many social factors are involved in suicidality that one does not require a mental health professional to be involved per se (e.g., early intervention to address substance use or unhelpful cognitions could prevent a problem from spiralling). However, the question of responsibility is a complex one in an industry typified by high levels of self-employment (134) and cultures of informality (135, 136). Undeniably, these complexities demonstrate how effective suicide prevention for musicians is likely to be best achieved by broad formulations and comprehensive initiatives encompassing the areas of professional life in which these musicians work as well as their wider social support networks.

### 3.3 Element 3: identifying

“Identify individuals with suicide risk via comprehensive screening and assessment” (115)

Risk among musicians is complex to identify. Although suicide risk stratification (i.e., low, medium, high) has been the standard of care for many years (137), a more sophisticated conceptualisation of suicide assessment has been developed by Pisani et al. (138) which may be more appropriate among musicians and music industry workers who experience inherent changes in risk status and risk state as well as the reduction in resources and potential for foreseeable changes (e.g., interpersonal difficulties) that often accompany their lifestyle. Evaluating this assessment in this population is crucial going forward. Likewise, although Franklin and colleagues (44) clearly identify risk factors for suicide, with musicians appearing to exhibit many, the researchers concluded that “prediction was only slightly better than chance,” suggesting that our ability to determine who will think about or consider suicide, engage in suicidal behaviours, or die by suicide – whether among musicians or otherwise – with predictive accuracy is not reliable (see (139) for more).

Nevertheless, certain warning signs specific to musicians may be identifiable based on an individual’s current psychological state (e.g., behaviours preparing for suicide), which suggest a proximal rather than distal association to suicidal behaviours (140), and indicate near-term risk (e.g., minutes, hours, or days); *risk* factors on the other hand suggest risk over a longer-term ranging from a year to a lifetime (141). Some examples of warning signs that may be particularly relevant to musicians include: withdrawing from people, engaging in risk-taking behaviours, increased use of alcohol and/or drugs, changes in sleeping and/or eating patterns, and writing about death in their lyrics. Ensuring that musicians themselves can identify these warning signs, as well as those around them in their personal and professional networks, in conjunction with a comprehensive understanding of more consolidated risk and protective factors over time may aid in risk level determination.

### 3.4 Element 4: engage

“Engage all individuals at-risk of suicide using a suicide care management plan” (115)

Clinician-led, ‘prevention-oriented’ risk formulation strategies - e.g., Pisani et al. (142) – to guide prevention planning may prove useful for an occupational group like musicians with varying risk states. The Columbia Suicide Severity Rating Scale (C-SSRS) Screen Version - a valid and reliable, six item measure designed to screen for the presence and severity of suicidal ideation and suicidal behaviour (143, 144) - followed by the C-SSRS clinical assessment and treatment protocol, as suggested by the Zero Suicide Initiative (145) and the Joint Commission (189), represent an effective evidence-based option to consider when attempting to determine suicide risk among musicians. However, the C-SSRS has not been evaluated among musicians, and further research evaluating the use of this instrument among this population is needed to enrich our understanding. In addition, the Safety Planning Intervention (SPI) (146) is an evidence-based and effective technique to reduce suicide risk which can be personalised to each high-risk individual, be developmentally and culturally appropriate, include strategies that can be used at all times of day or night, and list relevant helplines (e.g., the 1393 suicide prevention hotline in Korea). There has been research providing evidence for the effectiveness of the SPI (albeit not among musicians specifically) (147, 148), and it has been shown to be effective in reducing suicidal ideation and behaviour across high-risk populations either as a stand-alone intervention, which may be utilised via smartphone apps (149) – which may offer particular benefits for musicians with busy schedules on tour, for example - or combined with other prevention approaches.

Alongside this, it is suggested by the ZSF that limiting access to means is a core element of ‘engage’. In the case of popular musicians, a clear risk factor in early mortality is that of substance abuse and overdose as means for suicide. Chertoff and Urbine (150) have shown that popular musicians are more likely to die from alcohol and drug abuse than non-musicians and thus clinical strategies to reduce the access to lethal means, particularly among high-risk individuals, are an effective approach for preventing suicide. One such intervention known as lethal-means safety counselling is a patient-centred suicide prevention practice (151) which encourages potentially suicidal people and, crucially, their families to make suicide methods, including prescribed medications and illicit substances less available or more difficult to immediately access (152) [see recent systematic review (153)]. Among some musicians working in certain genres of popular music, therefore, having conversations about the dangers of substances and potential overdoses, both unintentional and intentional, may save lives. Counselling on Access to Lethal Means [CALM (154)], has recently been modified as a form of training for the general public (as opposed to solely healthcare professionals) and demonstrated promising results vis-à-vis increasing the comfort, confidence, and willingness to have a conversation about reducing access to lethal means in situations where individuals may be showing signs of suicide (155). Thus, awareness, education, and training to counsel musicians at risk for suicide and their loved ones to reduce access to lethal means should be considered as a prevention strategy.

### 3.5 Element 5: treat

“Treat suicidal thoughts and behaviours directly using evidence-based treatments” (115)

Research has demonstrated that interventions designed to directly address suicidality as opposed to other mental health constructs (e.g., depression or anxiety) may be more effective in decreasing suicidal thoughts and behaviours (156). With regards to suicide-specific interventions, there have been three evidence-based treatments that have been developed and shown to decrease suicide risk among a variety of populations. These include Dialectical Behaviour Therapy [DBT (157)], Cognitive Therapy for Suicide Prevention [CT-SP (158)], and the Collaborative Assessment and Management of Suicidality [CAMS (159)] (see Swift et al. (160) for a recent meta-analysis of CAMS compared with alternative treatments, indicating CAMS demonstrated significantly lower suicidal ideation and general distress, significantly higher treatment acceptability, and significantly higher hope/lower hopelessness). In sum, these three suicide-focused therapies should be employed with musicians and music industry workers who are experiencing suicidal ideation and/or have engaged in suicidal behaviour, and their use evaluated.

However, no recommendations are available for suicide treatment among musicians *specifically*. That being said, suicide intervention strategies have been developed for other groups with similar risk factors mentioned above [e.g., high levels of depression (25, 32)], which may be adapted for musicians. We will examine an intervention below targeted at groups (as per musicians) with an increased risk of suicide and suggest how it might offer insights which could be helpful for musicians who are at risk of suicide.

Cognitive Behavioural Therapy for Suicide Prevention [CBT-SP (161)] is an evidence-based treatment approach developed to reduce suicide risk through targeted interventions addressing cognitions and behaviours associated with suicidal crises developed, principally, for use with depressed and suicidal individuals. The therapy involves conducting detailed analyses of suicidal events to identify triggers, developing coping skills, and implementing means restriction strategies while accounting for the specific challenges patients may encounter (162). For musicians, CBT-SP may emphasise cognitive restructuring to address maladaptive thinking patterns or beliefs while also incorporating stress management techniques to cope with some of the forms of occupational stress outlined above. Additionally, CBT-SP may help musicians develop healthy routines and support networks despite irregular touring schedules, and can address industry-specific risk factors such as substance use (81) and financial instability (45). Research has shown that CBT-SP can be effectively adapted for specific populations while maintaining its core evidence-based principles (163), suggesting its potential utility for addressing suicide risk in the music community, something which requires examination in future empirical work. Given these findings, implementing culturally-informed CBT-SP interventions tailored to musicians’ unique needs and pressures could be a promising approach for reducing suicide risk in this vulnerable population.

In addition, CBT-SP has been shown to be suitable among student athletes who, like musicians, tend to exhibit perfectionist beliefs (164), but Flett et al. (165) suggest that “general prevention programs without an explicit or extensive focus on perfectionism are less successful.” However, there are no perfectionism-specific suicide treatments we can look to which might be applied to musicians. This presents a challenge given evidence concerning high levels of perfectionism in certain musicians (particularly those in the classical tradition) (166) and the links between perfectionism and suicide (167, 168). Indeed, for musicians building careers in societies known for high levels of socially prescribed perfectionism the risk of suicide may be even higher (169), as per our earlier discussion of K Pop in Korea. This represents an important area of further research given that, as Flett et al. (165) suggests, suicide treatment for perfectionism must be proactive given the tendency among perfectionists to conceal their suffering, and need to be specifically targeted at perfectionism as opposed to more general constructs.

Finally, the epidemiological data which began this paper reveals high levels of suicide by women in this occupational group relative to women in the general population. Therefore, mental health promotion and suicide prevention programs should be tailored specifically for women [see (170)], particularly women in the music industry. Given that women musicians may be experiencing a unique set of stressors (52), suicide preventative intervention programs should be developed and evaluated in this high-risk group.

### 3.6 Element 6: transitioning

“Transition individuals through care with warm hand-offs and supportive contacts” (115)

The transition component of the ZSF requires particular attention and adaptation for musicians given their unique lifestyle demands, and in our discussion we focus specifically on touring [see also (40, 60)], reiterating again that the opinions offered below have not been evaluated among musicians in the empirical literature to date. Care transitions must be carefully coordinated through warm hand-offs between providers (171), especially when musicians are touring across different locations. This may require establishing networks of providers in various cities or implementing telehealth options to maintain continuity of care (172). Providers should routinely use caring contacts (173), appointment reminders, and bridge appointments to ensure treatment adherence, particularly when musicians are on the road and potentially in an unstable environment. These evidence-based transition practices become especially important during high-risk periods such as post-tour depression or after negative career events.

To facilitate effective transitions, providers should create detailed crisis response plans (174) or safety plans (146) that account for touring schedules and varying access to care resources in different locations. These plans should identify local emergency services and support contacts in frequent tour destinations, while also incorporating virtual care options when in-person services are not feasible (175). Treatment teams should also coordinate with tour management and trusted support personnel to ensure proper implementation of crisis response and safety plans as well as consistent monitoring during transitions between providers and/or locations, following best practices for care coordination in suicide prevention (176). These coordinated transition efforts, when tailored to the unique demands of the music industry, may help ensure continuity of care and reduce suicide risk even as musicians navigate the challenges of touring and frequent travel.

### 3.7 Element 7: improving

“Improve policies and procedures through continuous quality improvement” (115)

Given the relative dearth of data on musician suicide, data must be collected in this high-risk and vulnerable group of individuals. This data-driven research should inform the development of interventions addressing the unique needs of musicians. Moreover, interventions need to be evaluated and refined to ensure the efficacy of the treatment approach. Organisations implementing the ZSF for musicians should establish clear metrics for tracking outcomes, including rates of screening, assessment, safety planning adherence, engagement in treatment, and successful care transitions during touring (171). This data collection should address industry-specific risk factors outlined thus far in this paper, such as: performance anxiety, perfectionism, touring stress, family conflict, substance use, and career instability. Quality improvement efforts should focus on identifying gaps in care delivery and adapting interventions based on musician feedback and outcomes data (177). This may include evaluating the effectiveness of telehealth interventions for touring musicians, assessing the impact of peer support programs within the music industry, and/or measuring the success of culturally-adapted safety planning approaches, and these findings should be peer reviewed. Additionally, providers should track disparities in care access and outcomes across different segments of the music industry, paying particular attention to underserved groups and emerging artists who may have access to fewer resources and specific stressors (178). Regular reviews of this data can guide refinements to suicide prevention protocols and inform best practices for supporting musicians’ mental health needs. By prioritising data-driven, tailored approaches, stakeholders can ensure that suicide prevention efforts within the music industry are both equitable, accessible, and effective, ultimately fostering a culture of mental health support that resonates with musicians’ unique challenges, lifestyles, and experiences.

## 4 Conclusion

In sum, suicide is a serious problem that has not been sufficiently addressed among those who pursue careers in the music industry. Routine mortality figures from the United Kingdom and the United States suggests that musicians in these territories are, despite important methodological challenges concerning categorisation, a demonstrably at-risk group for suicide. It also appears from our analysis that certain genres and geographical territories, such as K Pop in Korea, require further empirical work to better understand risk, prevention and intervention in this specific socio-political and cultural context. Indeed, better evidence concerning musicians of all kinds, working in all genres, with a global focus, is desperately needed. The paucity of evidence regarding risk factors in this occupational group underscores the need for comprehensive quantitative and qualitative research to inform the development and evaluation of targeted suicide prevention interventions. More work is needed to characterise risk factors for suicidal thoughts and attempts in this occupational group so that such risk factors might be mitigated in tailored suicide prevention approaches, evaluated for effectiveness and acceptability. Pending these evaluations, we can only suggest possible solutions, as we have sought to do. In applying the Zero Suicide Framework and adapting it for musicians (and the wider music industry), we have offered a comprehensive and multifaceted approach encompassing both individual and systemic preventative strategies to reduce the risk of suicide in this high-risk group, while acknowledging that these interventions have not yet been evaluated among musicians. This has encompassed elements of: leadership (campaigns to raise awareness and leading cultural conversations at the highest level about suicide risk in the music industry), training (suicide gatekeeper training and empowering those in musicians’ wider networks), identifying (understanding musician-specific risk factors and warning signs), engaging (C-SSRS, SPI, lethal-means safety counselling, and CALM), treating (DBT, CT-SP, CAMS, CBT-SP, and the need for perfectionism-specific treatments), transitioning (caring contacts, crisis response plans), and improving (the need for data-driven research).

Further investigation is warranted to elucidate their unique psychosocial stressors in order to design and evaluate interventions specifically for musicians. In addition, we also need to enhance public understanding of the difficulties faced by musicians as well as train clinicians working within the music industries in the provision of evidence-based screening, assessment, brief interventions, and suicide-specific treatments to improve mental health and suicide-related outcomes. Moreover, additional research (particularly qualitative) is needed to explore musicians’ experiences of suicide in order to inform the development of future initiatives. It is also important to acknowledge a limitation in that, as aforementioned, this paper is a cross-disciplinary position piece and is neither a systematic nor scoping review. However, we hope to have presented one of the first papers of its kind to bring together and critically review the small body of emerging evidence in this field, as well as make suggestions to aid in suicide prevention, among an occupational group who provide the music which profoundly enriches our lives as music-listeners. We should, likewise, take seriously the task of enriching their lives as music-makers.

## In memoriam

This paper is dedicated to country music singer Kyle Jacobs, K Pop singer Moonbin, trot singer Haesoo, singer songwriter Coco Lee, and rappers Chino XL and OG Maco, all of whom died by suicide in 2023 and 2024 when this paper was being prepared and reviewed.

## Footnotes

^1^For the avoidance of doubt, the phrase ‘popular music’, ‘popular musicians’, and/or ‘popular music industry/industries’ is used throughout this paper to refer to (musicians working in) non-classical genres of music, and does not relate to fame, eminence or success (however defined).

^2^SMR = 120 (107–135) for Culture, Media and Sports occupations. However, direct comparisons with other groups are challenging given that many of the sub-groups above are within other sub-major occupations (Table 3 in the ONS 2017 Excel data file).

^3^This has even been examined with reference to portrayals of suicide by musicians (179), for example musical biopics (180), and is also echoed in studies exploring the impact of the Werther effect in Korea following K Pop suicides (94, 181).

^4^It is important to note that some of the forms of suicide prevention intervention proposed here for use with musicians may already exist, but that information about their use is not in the public domain nor is peer-reviewed work about their effectiveness available.
